# Distinct Genetic Loci Control Plasma HIV-RNA and Cellular HIV-DNA Levels in HIV-1 Infection: The ANRS Genome Wide Association 01 Study

**DOI:** 10.1371/journal.pone.0003907

**Published:** 2008-12-24

**Authors:** Cyril Dalmasso, Wassila Carpentier, Laurence Meyer, Christine Rouzioux, Cécile Goujard, Marie-Laure Chaix, Olivier Lambotte, Véronique Avettand-Fenoel, Sigrid Le Clerc, Laure Denis de Senneville, Christiane Deveau, Faroudy Boufassa, Patrice Debré, Jean-François Delfraissy, Philippe Broet, Ioannis Theodorou

**Affiliations:** 1 JE2492, Faculty of Medicine Paris-Sud, Univ Paris-Sud, Villejuif, France; 2 CHU Pitié Salpetrière (AP-HP), INSERM U543, Université Pierre et Marie Curie, Paris, France; 3 INSERM, U822, Univ Paris-Sud, Faculté de Médecine Paris-Sud, AP-HP, Hopital Bicêtre, Epidemiology and Public Health Service, Le Kremlin-Bicêtre, France; 4 CHU Necker (AP-HP) EA 3620 Université Paris Descartes, Paris, France; 5 CHU Kremlin Bicêtre (AP-HP); INSERM, U802, Univ Paris-Sud, Faculté de Médecine Paris-Sud, Le Kremlin-Bicêtre, France; 6 Chaire de Bioinformatique, Conservatoire National des Arts et Métiers, Paris, France; 7 Assistance Publique-Hôpitaux de Paris (AP-HP), Hôpital Paul Brousse, Service de Santé Publique, Univ Paris-Sud, Villejuif, France; Institut Pasteur Korea, Republic of Korea

## Abstract

Previous studies of the HIV-1 disease have shown that HLA and Chemokine receptor genetic variants influence disease progression and early viral load. We performed a Genome Wide Association study in a cohort of 605 HIV-1-infected seroconverters for detection of novel genetic factors that influence plasma HIV-RNA and cellular HIV-DNA levels. Most of the SNPs strongly associated with HIV-RNA levels were localised in the 6p21 major histocompatibility complex (MHC) region and were in the vicinity of class I and III genes. Moreover, protective alleles for four disease-associated SNPs in the MHC locus (rs2395029, rs13199524, rs12198173 and rs3093662) were strikingly over-represented among forty-five Long Term HIV controllers. Furthermore, we show that the HIV-DNA levels (reflecting the HIV reservoir) are associated with the same four SNPs, but also with two additional SNPs on chromosome 17 (rs6503919; intergenic region flanked by the DDX40 and YPEL2 genes) and chromosome 8 (rs2575735; within the Syndecan 2 gene). Our data provide evidence that the MHC controls both HIV replication and HIV reservoir. They also indicate that two additional genomic loci may influence the HIV reservoir.

## Introduction

In the past ten years, candidate gene approaches were used to investigate host genetic variability in HIV-1 disease. Chemokine receptors such as CCR5 and CCR2 [1] and Major Histocompatibility Complex (MHC) class I genetic variation have been definitively associated with either clinical disease progression or HIV-RNA levels [2], [3]. Recent advances in large-scale genotyping technologies enabled genome-wide association (GWA) strategies for detection of novel genetic variants that influence HIV-1 disease. In this respect, the first GWA study in HIV-1 disease [4] identified three Single Nucleotide Polymorphisms (SNPs): two of them, linked with the ability to control plasma HIV-RNA levels, were located within the MHC region (rs2395029 within HCP5 gene and rs9264942 near HLA-C), while a third SNP (rs9261174), located near ZNRD1, was only associated with disease progression. These data suggest that disease progression and HIV-RNA might be controlled by several loci of the human genome.

Plasma HIV-RNA levels differ largely between patients. This is the best known virological marker of HIV disease progression; its predictive value was proven at different points of the time course of HIV infection [5]–[7]. HIV-DNA levels in peripheral blood monononuclear cells (PBMC) is another major predictive marker for HIV disease progression representing a phenotype not yet studied in either candidate gene or GWA approaches [8], [9]. HIV-DNA and HIV-RNA levels measure two different forms of HIV-1. It is not clear whether HIV-DNA levels measure only the viral reservoir. Indeed both phenotypes can be determined accurately and, even though they are correlated, they are distinct predictive factors for HIV disease progression. Indeed plasma HIV-RNA is a composite marker reflecting viral fitness, replicative capacity and host control, while intracellular HIV-DNA levels reflect the establishment of HIV reservoirs and, thereof, the effect of numerous host proteins that may interact with the intracellular viral lifecycle [10].

We hypothesized that genetic variants that regulate HIV-DNA metabolism, in particular HIV latency, are different than those involved in HIV-RNA metabolism and HIV replication. For this purpose, we conducted a GWA study in the large ANRS PRIMO cohort of unselected HIV-1 seroconverters (observed median time since infection: 46 days) for identifying SNPs associated with early plasma HIV-RNA levels[11]. All 605 Caucasian subjects of this cohort were genotyped using Illumina Sentrix Human Hap300 Beadchip containing 317,139 tagging SNPs. We selected genetic variants having a high likelihood of being associated with plasma HIV-RNA levels for a false discovery rate (FDR)[12] of 25%.

In order to provide additional evidence to support these selected HIV-1 disease-related SNPs, we compared their allelic frequencies between this reference population (PRIMO) and an independent population of long-term HIV controllers[13]. The latter group of patients represents less than 0.5% of the general population of HIV-1 infected patients, and reflects an extreme phenotype compared to the PRIMO population. HIV controllers exhibit sustained spontaneous control of viral replication after at least 10 years of HIV infection (observed median time since HIV diagnosis 18 years). We hypothesized that true positive genetic variants obtained from the GWA will exhibit enrichment for protective alleles in long-term HIV controllers compared to patients from the PRIMO population. We also studied for the first time association signals for another viral phenotype, i.e., cellular HIV-DNA levels.

Taken together, our data redefine the regions controlling HIV-RNA within the MHC locus and indicate an independent effect of SNPs located nearby not only class I but also class III genes. Some of these SNPs are associated with HIV-RNA levels from primary infection and early seroconversion and are over-represented in patients showing long-term spontaneous control. Most importantly, our data provide evidence that the MHC locus controls both HIV replication and HIV-DNA levels while early control of HIV-DNA levels may be under the control of two additional genomic loci.

## Results

### GWA and plasma HIV1-RNA levels

**Figure 1:**
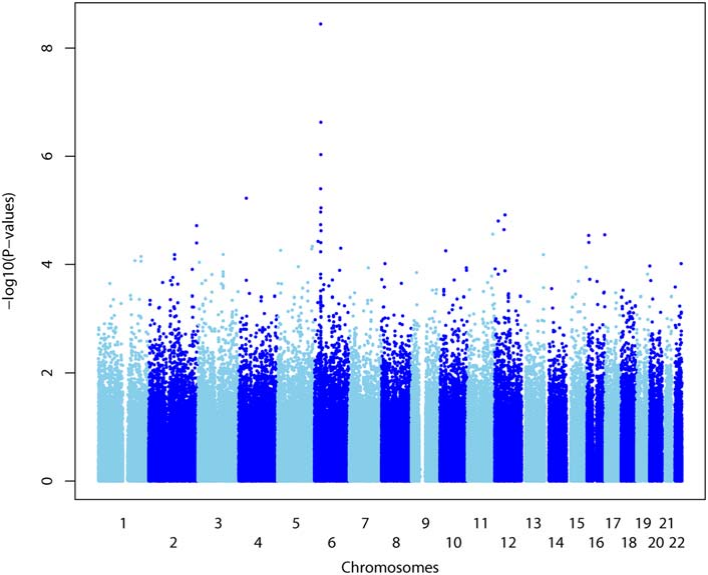
Genome wide association (GWA) study for plasma HIV-RNA levels in the ANRS PRIMO cohort. Negative log_10_ (P)-values for the score test for genome-wide association across the genome ordered from 1pter to 22qter. Adjacent chromosomes are shown in light blue and dark blue. The spacing between SNPs does not reflect actual physical distances. The false discovery rate was calculated based on a stratified approach (by chromosome) leading to chromosome-specific thresholds.

A total of 308,222 SNPs on the autosomal chromosomes passed the quality-control filters. We tested for association of genotype and early plasma HIV-RNA levels using a linear regression model assuming an additive genetic effect with adjustment for gender. The calculated log_10_ P-values for the SNPs genotyped in the 605 patients from the PRIMO cohort are plotted in Figure 1.

The SNPs associated with plasma HIV-RNA levels and selected for a FDR threshold of 25% are shown in Table 1; 15 SNPs were located on Chromosome 6, 3 each in chromosomes 12 and 16, and one on Chromosome 4. Among the 15 SNPs located in Chromosome 6, twelve were clustered within the 6p21 region harbouring the MHC (from 312 kb to 321 kb). The strongest association signal was observed for the rs10484554 SNP, which is located upstream from HLA-C. The rs10484554(T) protective allele had frequency of 0.128 in our population. The second highest association signal was observed for the rs2523619 SNP which lies within the HLA-B gene which is in strong linkage disequilibrium (r^2^ = 0.57) with the rs10484554 SNP. The third highest association signal obtained in the presented study was for the rs2395029 which lies within the class I gene HCP5 with weak linkage disequilibrium with rs10484554 (r^2^ = 0.13) and rs2523619 (r^2^ = 0.07). This association signal for rs2395029 replicates the result previously reported from CHAVI [4]. Another three HLA-C neighbouring SNPs (rs130065, rs3130467 and rs3130473) showing strong linkage disequilibrium (r^2^>0.5) and significant effects on viral load were among the top signals.

**Table 1:** The list of the most significant SNPs (FDR<25%) associated with plasma HIV-RNA levels during primary HIV-1 infection.

SNP	Gene/Region	Chr	Position	PRIMO Q-values	PRIMO P-values	Beta	CHAVI P-values	CHAVI rank*	MA	MA frequency in PRIMO	MA frequency in HIC	P-values
rs10484554	Intergenic HLA-C,HLA-B	6	31382534	7.45E−05	3.58E−09	0.533(T)	8.06E−07	2	T	0.128	0.222	1.30E−02
rs2523619	Intergenic HLA-C,HLA-B	6	31426123	2.46E−03	2.36E−07	−0.376(A)	6.33E−06	7	G	0.202	0.267	1.71E−01
**rs2395029**	**Intragenic HCP5**	**6**	**31539759**	**6.46E−03**	**9.32E−07**	**−0.916(T)**	**9.36E−12**	**1**	**G**	**0.026**	**0.167**	**4.24E−08**
rs130065	Intragenic CCHCR1	6	31230479	2.09E−02	4.01E−06	0.335(T)	3.57E−02	11217	T	0.181	0.279	4.01E−02
**rs13199524**	**Intragenic TNXB**	**6**	**32174743**	**3.74E−02**	**8.98E−06**	**0.487(T)**	**8.22E−04**	**295**	**T**	**0.080**	**0.222**	**4.58E−05**
rs3130467	Intergenic HCG27, HLA-C	6	31295054	3.74E−02	1.08E−05	0.302(T)	NA	NA	T	0.250	0.411	9.24E−04
rs3130473	Intergenic HCG27, HLA-C	6	31307187	5.49E−02	1.84E−05	0.302(T)	2.57E−03	877	T	0.217	0.356	2.95E−03
**rs12198173**	**Intragenic TNXB**	**6**	**32134786**	**6.23E−02**	**2.39E−05**	**0.458(A)**	**3.34E−04**	**149**	**A**	**0.083**	**0.222**	**6.54E−05**
rs214590	Intragenic AOF1	6	18317561	8.25E−02	3.75E−05	0.256(A)	2.06E−01	62534	A	0.432	0.356	1.60E−01
**rs3093662**	**Intragenic TNF**	**6**	**31652168**	**8.25E−02**	**3.96E−05**	**−0.412(A)**	**1.33E−04**	**75**	**G**	**0.091**	**0.261**	**1.19E−05**
rs2306942	Intragenic LAMA2	6	129677493	9.50E−02	5.02E−05	0.558(T)	3.42E−01	103217	T	0.048	0.033	6.18E−01
rs2894207	Intergenic HLA-C,HLA-B	6	31371730	1.02E−01	5.86E−05	−0.312(T)	1.82E−06	4	C	0.183	0.222	3.83E−01
rs1234818	Intergenic RPAP3, LOC728148	12	46251551	1.07E−01	2.29E−05	−0.247(A)	8.31E−01	249567	A	0.475	0.489	8.26E−01
rs3782478	Intragenic SCN8A	12	50354263	1.07E−01	1.22E−05	−0.258(T)	2.13E−01	64532	C	0.404	0.378	6.56E−01
rs978561	Intergenic LMO3,LOC390297	12	16948690	1.07E−01	1.58E−05	0.248(T)	6.55E−01	196348	T	0.472	0.378	1.06E−01
rs11725412	Intergenic TBC1D1, KLF3	4	37954149	1.10E−01	5.97E−06	0.580(A)	3.01E−01	90995	A	0.055	0.156	6.58E−04
rs2290902	Intragenic FAM38A	16	87310177	1.18E−01	2.83E−05	0.463(T)	5.56E−01	166828	T	0.076	0.080	1.00E+00
rs2313427	Intragenic ABAT	16	8722501	1.18E−01	2.92E−05	0.376(A)	1.31E−01	39978	A	0.106	0.068	3.10E−01
rs8050335	Intergenic C16orf72,L0C653737	16	9137278	1.18E−01	3.95E−05	0.341(A)	6.68E−01	200423	A	0.150	0.144	1.00E+00
rs6902462	Intragenic NKAIN2	6	124429862	2.06E−01	1.29E−04	−0.353(A)	9.19E−02	28165.5	G	0.098	0.078	6.10E−01
rs3869109	Intergenic HCG27,HLA-C	6	31292175	2.27E−01	1.52E−04	0.230(T)	8.41E−02	25766	T	0.369	0.467	2.22E−03
rs11967684	Intergenic HCG27,HLA-C	6	31307745	2.48E−01	1.79E−04	−0.212(A)	4.48E−02	14038	A	0.364	0.170	7.97E−04

The following information is given for each SNP: **SNP** is the rs number; **Chr** is the chromosome; **Position** is the position along the chromosome (Human Genome NCBI Build 35.1); **PRIMO Q-values** are the Q-values for the genome-wide association study with HIV-RNA in the Primo cohort; **PRIMO P-values** are the P-values for the genome-wide association study with HIV-RNA in the Primo cohort; **Beta** is the slope parameter (allele indicated in bracket is used as a reference group); **CHAVI P-values** are the P-values obtained from the CHAVI study; **CHAVI rank** is the rank of the p-values (obtained from the CHAVI study) according to the Illumina 317K whole-genome single-nucleotide polymorphism arrays. The rank assigned to tied values is calculated by taking the mean of the ranks. **MA** is the minor allele in the HapMap CEU population; **P-values** are the P-values for the comparison of allele frequencies between the PRIMO and HIV controllers patients (**HIC**). The four major SNPs (printed in bold) are those being significantly associated with plasma HIV-RNA and showing enrichment in HIV-controllers for protective allelic variants.

* NA = Not Available (SNP discarded in the Chavi study).

When comparing allelic frequencies between HIV-controllers and the PRIMO seroconverters, four SNPs located within the 6p21 (rs2395029, rs13199524, rs12198173 and rs3093662, are in bold in Table 1), showed highly significant differences (p<10^−4^) that correspond to a striking enrichment in HIV-controllers for protective allelic variants associated with HIV-RNA levels. We further designated them as major SNPs. Moreover, these four major SNPs showed highly significant p values (p<10^−7^) in the CHAVI study (Table 1). It should be noted that our study on the PRIMO cohort used plasma HIV-RNA levels measured at the time of primary infection, while the CHAVI study used viral loads measured during the plateau phase (6 to 18 months following seroconversion). Overall these results support the idea that these major SNPs have an effect on viral load throughout the whole time course of HIV disease.

**Figure 2:**
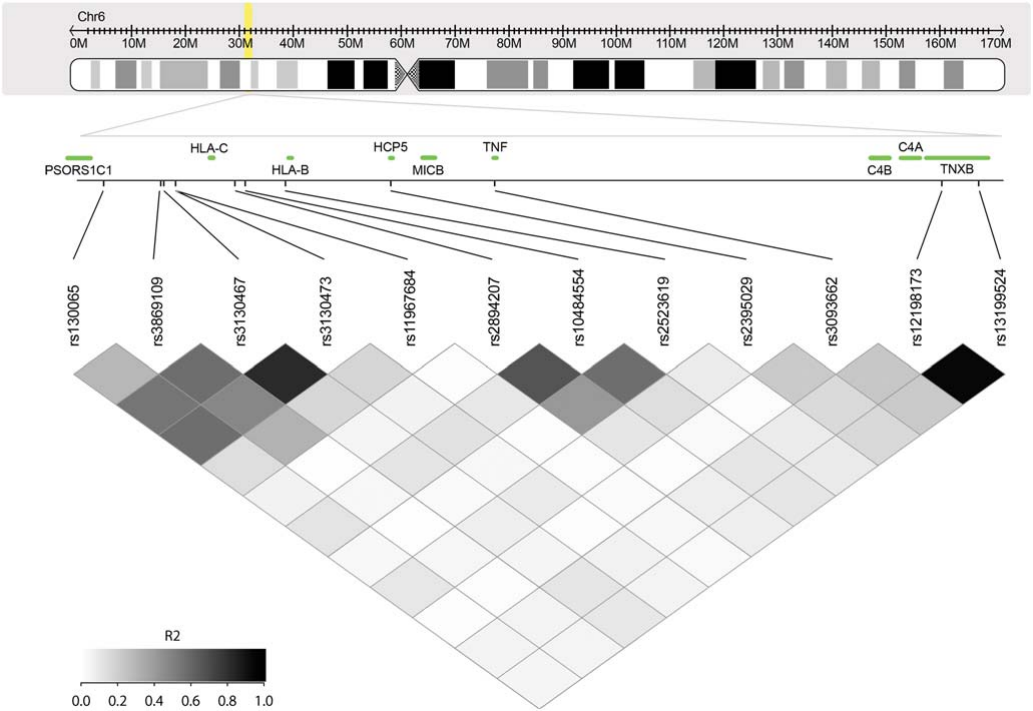
Location of the twelve selected SNPs in the MHC region (6p21) and linkage disequilibrium plot. Upper box shows an ideogram depicting chromosome 6 and the annotated region (transparent yellow rectangle). Lower box shows the linkage disequilibrium data matrix calculated from the European HapMap population. The colour intensity of each box is proportional to the strength of the LD property (r^2^) for the marker pair.

The extent of linkage disequilibrium between selected SNPs (Table 1) lying in the 6p21 area is shown in Figures 2, and Figure S1 and Figure S2. The genomic region flanked by these SNPs contains both MHC class I and class III genes and extends from the PSORS1C1 gene to TNXB gene. Supplementary Figure 1 and 2 display the linkage disequilibrium data matrix (based on HapMap CEU data) and the genes located witin this 6p21 region (from 312 kb to 321 kb).

Among the class I genes, only HLA-C and HLA-B genes are adjacent to SNPs associated with the control of plasma viral load (Figure 2). It should be noted that neither the PRIMO (Table 1) nor the CHAVI studies[4] provide evidence for association signals with HLA class II. Among the major SNPs, the rs2395029 is in moderate linkage disequilibrium (r^2^ = 0.21) with the rs3093662 SNP which lies within the Tumor Necrosis Factor (TNF) gene. The rs12198173 and rs13199524 SNPs showed moderate linkage disequilibrium with SNP rs2395029 as well as with all the other SNPs in the class I MHC region. Finally the rs12198173 and rs13199524 SNPs are in nearly complete linkage disequilibrium (r^2^ = 0.97), are both located in the class III region of MHC and both lie within introns of the TNXB gene. Although mutations in the TNXB gene are responsible for the Ehlers-Danlos Syndrome[14,14], it is unlikely that this gene is responsible for the observed effect in HIV-1 plasma viral load. Other neighbouring genes, particularly C4A and C4B (which show different types of polymorphisms such as duplications, gene recombinations, insertions, and SNPs inducing the appearance of premature stop codons) are located in the immediate telomeric side of TNXB and linked both to autoimmune and infectious diseases and are excellent candidate genes for this effect [15]. These results provide evidence that the region encompassing the HCP5 and MIC genes as well as the class III region encompassing TNXB and C4 genes region both contribute to the control of HIV-RNA levels.

**Figure 3:**
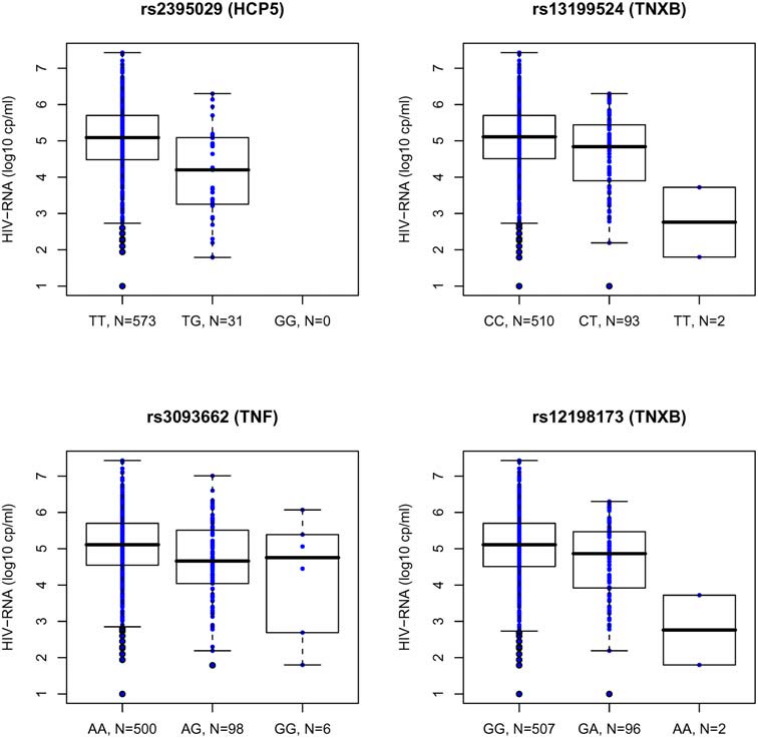
Boxplots for HIV-RNA levels in the PRIMO cohort for four major SNPs. Plasma HIV-RNA level is highly correlated with the rs2395029 (HCP5), rs3093662 (TNF), rs13199524 and rs12198173 (TNXB) genotypes.

Box plots for HIV-RNA levels measured during primary infection for the four major SNPs are shown in Figure 3. In patients homozygotes for the rs2395029(T) allele, the median value HIV-RNA was 5.09 (range: 4.48–5.70) whereas in heterozygous patients, the median was 4.20 (range: 3.25–5.09), illustrating that the minor allele G is associated with lower HIV-RNA levels. A similar trend for the minor allele G was observed for the rs3093662. Despite a minor allelic frequency of 0.083 (rs12198173) and 0.080 (rs13199524), with few observed homozygote patients for the protective alleles (A for rs12198173, T for rs13199524), a strong reduction (2.35 log_10_) for the HIV-RNA median level was observed. Both SNPs (rs12198173 and rs13199524) were located within the TNXB gene but the two homozygote patients also carried one favourable HLA allele such as HLA-B27 or HLA-B57. It should be noted that the homozygote patients for these protective alleles (rs12198173(A), rs13199524(T)) account for 4% of the HIV controllers versus 0.3% of the PRIMO seroconverters.

Another important result of this study was the strong association of the rs11725412 SNP located on chromosome 4 with plasma HIV-RNA levels both in the PRIMO and HIV controller cohorts (Table 1). Although we cannot exclude that this finding represents a false positive signal, the p-value on the PRIMO analysis was low (p<6×10^−6^) and a significant (p<6.58×10^−4^) enrichment was observed in HIV-controllers. The rs11725412 SNP lies in an intergenic region in 4p14, its immediate functional neighbours are the TBC1D1 gene (telomeric) and the KLF3 gene (centromeric). TBC1D1, the founding member of a family of proteins sharing a 180 to 200 amino acid TBC domain, is presumed to have a role in regulating cell proliferation and differentiation[16]. The KLF3 protein has a characteristic zinc finger domain and is classified in the family of SP transcription factors but its precise function remains unknown[17], [18]. More distant genes in this region include several Toll like receptors (TLR1,6,10) and PTTG2 (a gene belonging to the securin family) that plays an important role in cell division and survival[19], [20].

### GWA and cellular HIV-DNA levels

HIV-1 DNA levels in PBMCs are established at a very early stage of HIV infection and are representative of the HIV reservoir[21]. This marker showed a wide range (median = 3.30 log_10_ copies/millions PBMC; IQR = 0.76) in patients from the PRIMO cohort; in contrast, HIV-1 DNA levels were very low (median = 1.77 log copies/millions PBMC; IQR = 0.64) in long-term HIV-controllers. We hypothesized that this phenotype may be controlled by genomic regions different from those controlling HIV replication, particularly in phases of pre-integration or in provirus transcription that may be variable among patients.

The SNPs associated with HIV-DNA levels in the PRIMO cohort and selected for a FDR threshold of 25% are shown in Table 2. Among the 28 SNPs, 21 were located in the chromosome 6 (mainly in 6p21), 4 in chromosome 8, 1 in chromosome 17, 1 in chromosome 22 and 1 in chromosome 12. Some of these hits might represent false positives. However the four major SNPs (i.e., rs2395029, rs13199524, rs12198173 and rs3093662 are in bold in Table 2) previously found to be associated with HIV-RNA levels were also associated with HIV-DNA levels. This highlights the importance of the MHC region for both HIV replication and HIV reservoir. The first three SNPs (with q-value <2.3%, see Table 2) are located within three different chromosomes, specifically chromosomes 6, 17 and 8. The strongest association signal was observed for the rs2395029, one of the four major SNPs associated with plasma HIV-RNA (Table 1)[4]. The second highest association signal corresponds to rs6503919 SNP (chromosome 17). This SNP is found in an intergenic region flanked by DDX40[22] (which is a member of the DExH/D box family of ATP-dependent RNA helicases) (for a recent review see[23]), and YPEL2 (which is a putative zinc-binding protein gene)[24]. The rs2575735 SNP (chromosome 8) lies within the SDC2 gene which encodes for syndecan 2. Syndecan 2 is a trans HIV receptor which binds to HIV-1 gp120 through heparan sulfate chains[25], [26]. Keeping in mind that HIV controllers were selected on the basis of a specific HIV-RNA phenotype (<400 copies/ml after 10 years of known HIV infection), we observed no significant enrichment (p>0.1) for the protective alleles of rs6503919 and rs2575735 SNPs in HIV controllers compared to the PRIMO patients. Nevertheless, we cannot rule out the possibility that some of non-chromosome 6 hits may represent false positives.

**Table 2:** The list of the most significant SNPs (FDR<25%) associated with cellular HIV-DNA levels during primary HIV-1 infection.

SNP	Gene/Region	Chr	Position	Q-values	P-values	Beta	MA	MA frequency in PRIMO	MA frequency in HIC	P-values
**rs2395029**	**Intragenic HCP5**	**6**	**31539759**	**1.40E−02**	**6.72E−07**	**−0.540(T)**	**T**	**0.026**	**0.167**	**4.24E−08**
rs6503919	Intergenic YPEL2,DH40	17	54900747	1.66E−02	2.00E−06	−0.211(A)	G	0.174	0.233	1.66E−01
rs2575735	Intragenic SDC2	8	97603827	2.32E−02	1.34E−06	−0.176(A)	G	0.313	0.346	6.22E−01
rs4872550	Intragenic PEPB4	8	22665715	1.34E−01	2.31E−05	0.286(T)	T	0.069	0.044	3.98E−01
rs7013424	Intragenic PEPB4	8	22653259	1.34E−01	1.99E−05	−0.169(A)	G	0.267	0.222	3.78E−01
rs10484554	Intergenic HLA-C,HLA-B	6	31382534	1.47E−01	1.82E−05	0.225(T)	T	0.128	0.222	1.30E−02
rs130065	Intragenic CCHCR1	6	31230479	1.47E−01	4.26E−05	0.173(T)	T	0.181	0.279	4.01E−02
**rs13199524**	**Intragenic TNXB**	**6**	**32174743**	**1.47E−01**	**5.70E−05**	**0.255(T)**	**T**	**0.080**	**0.222**	**4.58E−05**
rs1524738	Intergenic Corf57,SMAP1	6	71381444	1.47E−01	6.91E−05	0.134(T)	G	0.443	0.430	8.27E−01
**rs3093662**	**Intragenic TNF**	**6**	**31652168**	**1.47E−01**	**2.18E−05**	**−0.247(A)**	**G**	**0.091**	**0.261**	**1.19E−05**
rs3095227	Intergenic MICB, MCCD1	6	31598979	1.47E−01	6.03E−05	0.156(T)	T	0.240	0.433	1.32E−04
rs3130637	Intergenic MICB, MCCD1	6	31596124	1.47E−01	6.35E−05	0.156(T)	T	0.240	0.433	1.30E−04
rs3130931	Intragenic POU5F1	6	31242867	1.47E−01	4.91E−05	0.149(A)	A	0.274	0.467	2.54E−04
rs9484823	Intergenic MCHR2, SIM1	6	100768057	1.47E−01	7.04E−05	0.174(A)	G	0.184	0.144	3.99E−01
rs3093993	Intergenic MICB, MCCD1	6	31598704	1.52E−01	8.01E−05	−0.154(T)	G	0.240	0.433	1.33E−04
rs4819648	Intergenic MICAL3, PEX26	22	16773564	1.66E−01	3.11E−05	−0.188(T)	T	0.160	0.156	1.00E+00
rs2313168	Intragenic PEBP4	8	22662063	1.73E−01	3.97E−05	0.288(A)	A	0.063	0.044	5.14E−01
rs12313095	Intergenic RBM19, TBX5	12	112931243	1.99E−01	1.33E−05	−0.156(A)	A	0.383	0.311	2.03E−01
**rs12198173**	**Intragenic TNXB**	**6**	**32134786**	**2.05E−01**	**1.28E−04**	**0.240(A)**	**A**	**0.083**	**0.222**	**6.54E−05**
rs2841307	Intergenic MCHR2, SIM1	6	100863408	2.05E−01	1.25E−04	−0.176(A)	A	0.154	0.133	6.57E−01
rs4140483	Intergenic MCHR2, SIM1	6	100799729	2.08E−01	1.49E−04	−0.176(T)	T	0.155	0.133	6.51E−01
rs9459971	Intergenic DLL1, TCTE3	6	170344288	2.08E−01	1.50E−04	−0.146(A)	G	0.263	0.250	8.03E−01
rs9321981	Intergenic MCHR2, SIM1	6	100839000	2.09E−01	1.61E−04	0.175(A)	G	0.155	0.133	6.52E−01
rs4321841	Intergenic NHSL1, CCDC28A	6	139118463	2.35E−01	1.99E−04	0.125(T)	T	0.493	0.455	5.12E−01
rs654690	Intergenic TAGAP, FNDC1	6	159434766	2.35E−01	2.03E−04	0.134(A)	A	0.342	0.389	4.18E−01
rs130072	Intragenic CCHCR1	6	31220463	2.37E−01	2.25E−04	−0.193(A)	A	0.108	0.044	8.00E−02
rs2073724	Intragenic TCF19	6	31237686	2.37E−01	2.28E−04	−0.194(T)	T	0.106	0.044	7.97E−02
rs7738698	Intergenic MTHFD1L, AKAP12	6	151599747	2.41E−01	2.43E−04	0.158(T)	C	0.182	0.189	8.90E−01

**SNP** is the rs number; **Chr** is the chromosome; **Position** is position along the chromosome (Human Genome NCBI Build 35.1); **Q-values** and **P-values** for the genome-wide association study with HIV-DNA in the Primo cohort are shown; **Beta** is the slope parameter (allele indicated in bracket is used as a reference group); **MA** is the minor allele in the HapMap CEU population; **P-values** for the comparison of allele frequencies between the PRIMO and HIV controllers patients (**HIC**) are shown. The four major SNPs are in bold.

**Figure 4:**
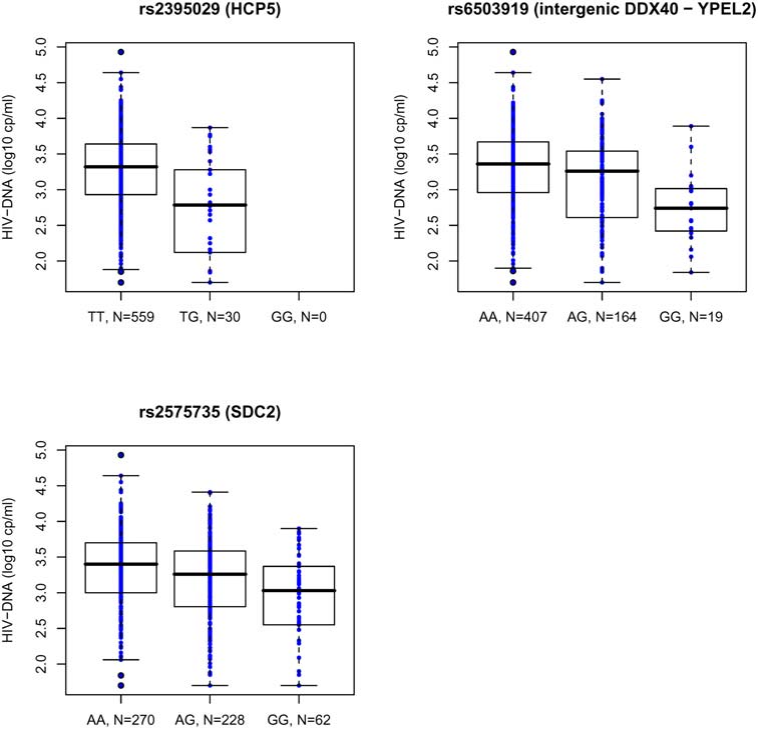
Boxplots for HIV-DNA levels in the PRIMO cohort for the three major SNPs. Cellular HIV-DNA level is highly correlated with the rs2395029 (HCP5), rs6503919 (intergenic DDX40 – YPEL2) and rs2575735 (SDC2) genotypes.

Box plots for the HIV-DNA levels in the PRIMO seroconverters for the first three SNPs (rs2395029, rs6503919, and rs2575735 as listed in Table 2) are shown in figure 4. The difference in median HIV-DNA levels between TT homozygotes and TG heterozygous patients for rs2395029 was −0.53, illustrating that the minor allele G is associated with lower HIV-DNA levels. A similar trend, i.e., a 0.36 difference in median values in HIV-DNA according to the genotype was also observed among HIV controllers. For the SNP rs6503919 located on chromosome 17, we observed both a −0.62 log_10_ difference in median HIV-DNA values between AA and GG patients in the PRIMO cohort, and a −0.45 log_10_ difference in HIV controllers according to the same genotype. A −0.62 log_10_ HIV-DNA difference is clinically highly significant since it can be compared to the effect obtained within the first year following antiretroviral treatment initiation with a classical triple therapy[27]. For the rs2575735 SNP in chromosome 8, we observed a reduction of −0.37 log_10_ between homozygotes AA and GG in the PRIMO patients. In contrast such a difference was not observed in HIV controllers. This result may imply a temporal effect which disappears over time.

## Discussion

The present GWA study identified SNPs highly associated with early viral replication in the ANRS PRIMO cohort. Although in this study, we performed the genotyping using an Illumina 317K chip, and gains in terms of genetic coverage and transferability[28] could be obtained using more recent technologies it should be noted that the top SNPs associated with the control of viral load are located in the MHC locus in our and Fellay's study even that the Fellay *et al.* study considered a SNP-chip having higher genetic coverage (Illumina 550K SNP). Furthermore the definition of the viral phenotype is different between Fellay's study and this study; further explaining differences in the power of associations. Four of these SNPs exhibited a striking enrichment of their protective allele in long-term HIV controllers as compared to the unselected seroconverters from the PRIMO cohort. These four major SNPs are located in the 6p21 MHC in the vicinity of class I and III genes. The association signal observed for the rs2395029 SNP located in the HCP5 gene, which is in complete linkage disequilibrium with HLA-B*5701[29], replicates the previous report from the Chavi study[4]. Interestingly, the rs2395029*T allele which has a protective role in HIV infection was also recently shown to be associated with increased susceptibility to psoriasis and psoriatic arthritis[30]. One possible explanation for this MHC association signal is that variants of the classical HLA-B and HLA-C alleles optimally present HIV-derived peptides to CD8+ T-cells which, in turn, kill HIV-infected cells. The results of the present study make an additional contribution to reports in the literature regarding the importance of HLA-B27 and HLA-B57 in long-term non-progression and in viral load control[31]–[33]. Another possible explanation relates to other MHC-related gene variants in linkage disequilibrium with rs2395029 such as SNPs in the TNF gene. The fact that the MICB gene is flanked by rs2395029 and rs3093662 SNPs also suggests its contribution to the control of HIV-1 RNA. MICs are polymorphic[34], [35] and affect cell-mediated cytotoxicity through interaction with NKG2D[36], [37] while TNF variants affect numerous steps of the immune response[38]. It is, thus, possible that HIV-infected patients who have a particular set of the classical either HLA-B or HLA-C alleles associated with TNF and MIC protective alleles have an increased capability to control HIV replication during the time course of HIV disease.

In addition, the present study also highlights that some of the major SNPs located in the MHC region have an effect on HIV-RNA levels independently of SNP rs2395029. The two major SNPs rs12198173 and rs13199524, which are in nearly complete linkage disequilibrium, suggest that the class III region of MHC plays an important role in HIV replication. In homozygote patients for the minor allele of these SNPs, plasma HIV-RNA was 2.35 log_10_ lower than in patients with other genotypes. To the best of our knowledge, the present is the first study demonstrating that a rare genetic variant within the class III region of MHC is associated with important viral load changes, which are comparable to that obtained during the first weeks following multiple antiretroviral therapy initiation. Undoubtedly, such an efficient control of viral replication, both in terms of strength and duration, provides new insights into underlying mechanisms and may lead to novel methodologies for HIV vaccine design.

Moreover, the present study highlights the importance of SNPs adjacent to HLA-C such as the rs10484554 which was the most highly associated SNP in our study. HLA-C has a dual function: it presents HIV-derived peptides to T lymphocytes but also interacts with either activatory or inhibitory NK receptors. Unfortunately, very few studies have focused attention on the role of HLA-C alleles in HIV disease[39]. Recent reports[40] provided evidence of epistatic interactions between classical HLA-B alleles and KIR-receptor polymorphisms but again only the role of HLA-B allelic variants was studied. It is worth noting that the protective rs10484554*T allele was also recently shown to be associated with increased susceptibility to psoriasis and psoriatic arthritis[30]. These findings suggest that HLA-C deserves more attention as an HIV disease modifying gene.

The present one is the first study which explored the role of genetic variants on the early establishment of the HIV reservoir. Association signals for HIV-DNA levels revealed new genetic loci that may control early steps of HIV spreading. The four major SNPs associated with viral replication were also found to be strongly associated with HIV-DNA levels. The strongest association signal for HIV-DNA was again observed for the rs2395029 SNP located in the HCP5 gene suggesting that structural variants of MHC genes also play a very important role on HIV reservoir.

Moreover, we found an association signal for the SNP rs6503919 in chromosome 17 which lies in an intergenic region flanked by the DDX40 and YPEL2 genes. One plausible explanation for this finding is that the Helicase DDX40 structural variants play an important role in the metabolism of HIV-RNA and/or in the efficiency of provirus transcription. DEAD helicases are generally thought to participate in many aspects of RNA metabolism including RNA transcription, mRNA export, translation and RNA stability. It is now well established that HIV-1 has the ability to use more than one cellular RNA helicases for its replicative life cycle (for a recent review see [23]). The other immediate neighbour gene of SNP rs6503919 is YPEL2, which is a member of the zinc-binding protein family. Besides the fact that it is associated with the mitotic apparatus, details of the exact functions of YPEL2 are not well known to date. It is possible that structural variants of YPEL2 affect the rate of division of immune competent cells, thus, limiting expansion of latently infected cells and their spreading to lymphoid tissues.

We also report an association signal on chromosome 8 for the rs2575735 SNP lying within the Syndecan 2 gene. The role of Syndecans (which are trans HIV receptors) on the HIV reservoir has not been investigated to date. Syndecans play a role for HIV dissemination to T lymphocytes and macrophages[25], [26]. Our findings support the idea that structural variants of Syndecans may be important in controlling HIV spreading and early establishment of the HIV reservoir. It should be noted that the protective variant was not over-represented in long-term HIV controllers, suggesting either a temporal effect or a lack of power due to the small number of HIV controllers tested. It is also important to keep in mind that the HIV controllers in the present study were selected on an HIV-RNA based phenotype (<400 copies/ml) but not on an HIV-DNA based phenotype. Further studies are necessary to confirm the role of Syndecan 2 genetic variants on HIV reservoirs.

Taken together our data provide evidence that four major SNPs in the MHC locus are associated with both control of plasma HIV-RNA and cellular HIV-DNA levels. Most importantly they are associated with viral load replication at the time of primary infection, at the viral set point but also at late stages of HIV-1 disease. It is worth noting that here we performed the genotyping using an Illumina 317K chip and that gains in terms of genetic coverage and transferability could be obtained using more recent technologies [28]. In our study, we provide evidence that the host genetic control on HIV-RNA over time is under the control of both MHC class I and class III genes. Finally, this is the first GWA study which investigated the host determinants controlling the HIV-1 reservoir and identified two new loci in different chromosomes; this result implies that additional and/or different genes than those controlling HIV replication control the establishment of the HIV reservoir.

## Materials and Methods

### Patients

Since November 1996, the ongoing French ANRS PRIMO CO 06 Cohort has enrolled patients during primary HIV-1 infection in 81 French hospitals[11]. The present study was approved by the Paris Cochin Ethics Committee. All subjects gave their informed written consent. Recent infection was confirmed by one of the following: an incomplete Western Blot pattern (absence of anti-p68 and anti p34); a detectable plasma HIV-RNA or positive p24 antigenemia a negative or weakly reactive ELISA test; an interval less than 6 months between an authenticated negative and a positive ELISA test. The date of infection was estimated on the basis of one of the following: the date of symptom onset minus 15 days, the date of the incomplete Western Blot minus one month, or the mid-point between a negative and a positive ELISA test. The interval between the estimated date of infection and enrolment did not exceed 6 months (median observed interval in the cohort = 46 days). Patients had to be antiretroviral-naïve at enrolment. Clinical and biological examinations were conducted at enrolment and at each follow-up visit, and included CD4 cell and plasma HIV-RNA assays. Blood cells and plasma were drawn at enrolment and at follow-up visits and frozen until analyses.

A total of 605 Caucasian patients were selected and genotyped on the ANRS Platform; in 590 of these patients intracellular HIV-DNA levels at enrolment were also measured. The patients were predominantly (87.8%) males and predominantly (96.0%) infected through sexual contact; their median age at enrolment was 34 years (IQR: 29–41).

Since May 2006, the ongoing French ANRS EP36 study has enrolled HIV controllers, defined as patients who had more than 90% of their viral loads <400 copies/ml after 10 years of known HIV infection, and were disease symptom-free and antiretroviral-therapy free. This study was approved by the Paris Bicêtre Ethics Committee. Patients were identified in hospitals throughout France, and were enrolled after written informed consent[13], [41]. At enrolment, 10 ml of whole blood were collected and frozen for further virological and genetic analyses. A total of 45 Caucasian HIV controller patients with available DNA samples were selected for this study.

### HIV-RNA and HIV-DNA measurements

Plasma HIV-RNA levels and HIV-DNA in PBMCs used in this paper were quantified in the first patient samples drawn at the time of enrolment in the PRIMO cohort and the HIV controllers study, prior to any antiretroviral therapy initiation. Median time from infection to enrolment was 47 days IQR 37–69 HIV-RNA and HIV-DNA levels measured during the 0–6 months following infection predict HIV disease progression [8]. HIV-RNA levels measured at the time of primary infection are strongly associated with HIV-RNA levels at the set point [42].

Quantification of HIV-RNA in plasma was performed on site at the participating hospitals, while HIV-DNA quantification was performed at the Necker Virology laboratory using real time PCR technology based on amplification of the HIV-1 LTR gene[43]. Briefly, after nucleic acid extraction, the total amount of DNA was quantified. The 95% detection threshold of the assay was 5 HIV-DNA copies/PCR, that is, 70 copies of HIV-DNA per million PBMC when using 0.5 µg of total DNA per PCR. For HIV controller samples, quadruplicate tests were conducted by PCR in order to get numerical data values for very low levels. Results were expressed as the log_10_ copies number per million PBMC.

### Illumina SNP genotyping and quality-control filtering

The DNA samples from all patients were genotyped with Illumina HapMap300 Beadarrays containing 317,000 SNPs. The scanned images were processed using BeadStudio (version 3.2.23) and all genotypes were called using the Illumina proprietary algorithm with genotype clusters generated from the series of samples tested.

Samples with call rate below 97% were discarded and SNPs with Gene Call<90% were removed. SNP with either a minor allele frequency below 0.01 or significant (p<10^−7^) deviation from Hardy-Weinberg equilibrium were excluded. After applying these filters, the average call rate across these samples was 98.3% with 308,222 SNPs on the autosomal chromosomes.

### Statistical analysis

#### Population stratification

To detect, and control, possible population stratification, we employed the genomic control approach[44] using all SNPs to estimate the genomic-control inflation factor λ.

The value for λ was small (λ = 1.005) indicating no population substructure in our cohort and inducing no inflation of the test statistics under the null hypothesis. We, therefore, reported all our analyses results without regard to specific treatment for population structure.

#### Multiple testing

To address the multiple testing problem, we selected SNPs considering a 25% level for the FDR which is the expected proportion of false discoveries among all discoveries[12]. The *q-values*, which are analogous to the adjusted P-values for the FDR criterion, measure the minimum FDR that is incurred when calling that test significant. In the present study, we estimated the *q-values* using the procedure introduced by Dalmasso *et al.* [45] and considering a stratified approach (by chromosome) as proposed by Sun *et al* [46].

#### Comparison between PRIMO and HIV controller patients

We compared allele frequencies between the PRIMO and HIV controller patients using an algorithm dedicated to association studies using bi-allelic markers and allowing fast computation of unbiased exact P-values[47].

#### Assessment of linkage disequilibrium between SNPs

To assess the level of linkage disequilibrium between SNPs, we calculated the pairwise r^2^ measure between consecutive pairs of markers throughout the genome using the expectation-maximization algorithm to estimate 2-locus haplotype frequencies which is implemented in the R package snpMatrix [48].

#### Genome wide association tests

We tested for an association between SNPs and HIV-RNA and HIV-DNA levels according to a linear model with an additive genetic effect with adjustment for gender. Significance was determined using generalized score tests [49] with SNP genotypes as the dependent variables and either HIV-DNA or HIV-RNA levels as the independent variables.

## Supporting Information

Figure S1 Upper box: An ideogram depicting chromosome 6 and the annotated region (transparent red rectangle). Middle box: show the SNPs with their associated -log10(P)-values, these lines are spaced according to their actual physical location. Positions of the four SNPs (rs2395029, rs13199524, rs12198173 and rs3093662) are indicated on the figure. Lower box: Linkage disequilibrium data matrix is based on HapMap CEU data. The figure has been constructed using WGAViewer software (http://people.genome.duke.edu/~dg48/WGAViewer/download.php, Ge D, Zhang K, Need AC, Martin O, Fellay J, Urban TJ, Telenti A, Goldstein DB. WGAViewer: software for genomic annotation of whole genome association studies.Genome Res. 2008, 18:640–643).
(0.54 MB TIF)

Figure S2 Upper box shows the SNPs with their associated -log10(P)-values, these lines are spaced according to their actual physical location. Positions of the four SNPs (rs2395029, rs13199524, rs12198173 and rs3093662) are indicated on the figure. Lower box shows the position of the UCSC known Genes (June, 05 and based on UniProt, RefSeq, and GenBank mRNA) located in the annotated region. The figure has been constructed using Genome Browser software, Kent WJ, Sugnet CW, Furey TS, Roskin KM, Pringle TH, Zahler AM, Haussler D. The human genome browser at UCSC. Genome Res. 2002,12: 996–1006).
(0.42 MB TIF)

## References

[1] Ioannidis JP, Rosenberg PS, Goedert JJ, Ashton LJ, Benfield TL (2001). Effects of CCR5-Delta32, CCR2-64I, and SDF-1 3′A alleles on HIV-1 disease progression: An international meta-analysis of individual-patient data.. Ann Intern Med.

[2] Kaslow RA, Carrington M, Apple R, Park L, Munoz A (1996). Influence of combinations of human major histocompatibility complex genes on the course of HIV-1 infection.. Nat Med.

[3] Gao X, Bashirova A, Iversen AK, Phair J, Goedert JJ (2005). AIDS restriction HLA allotypes target distinct intervals of HIV-1 pathogenesis.. Nat Med.

[4] Fellay J, Shianna KV, Ge D, Colombo S, Ledergerber B (2007). A whole-genome association study of major determinants for host control of HIV-1.. Science.

[5] Mellors JW, Rinaldo CR, Gupta P, White RM, Todd JA (1996). Prognosis in HIV-1 infection predicted by the quantity of virus in plasma.. Science.

[6] Hubert JB, Burgard M, Dussaix E, Tamalet C, Deveau C (2000). Natural history of serum HIV-1 RNA levels in 330 patients with a known date of infection. The SEROCO Study Group.. AIDS.

[7] Mellors JW, Rinaldo CR, Gupta P, White RM, Todd JA (1996). Prognosis in HIV-1 infection predicted by the quantity of virus in plasma.. Science.

[8] Rouzioux C, Hubert JB, Burgard M, Deveau C, Goujard C (2005). Early levels of HIV-1 DNA in peripheral blood mononuclear cells are predictive of disease progression independently of HIV-1 RNA levels and CD4+ T cell counts.. J Infect Dis.

[9] Kostrikis LG, Touloumi G, Karanicolas R, Pantazis N, Anastassopoulou C (2002). Quantitation of human immunodeficiency virus type 1 DNA forms with the second template switch in peripheral blood cells predicts disease progression independently of plasma RNA load.. J Virol.

[10] Brass AL, Dykxhoorn DM, Benita Y, Yan N, Engelman A (2008). Identification of host proteins required for HIV infection through a functional genomic screen.. Science.

[11] Desquilbet L, Goujard C, Rouzioux C, Sinet M, Deveau C (2004). Does transient HAART during primary HIV-1 infection lower the virological set-point?. AIDS.

[12] Benjamini Y, Hoschberg Y (1995). Controlling the false discovery rate:a practical and powerful approach to multiple testing.. J R Stat Soc Ser B.

[13] Lambotte O, Boufassa F, Madec Y, Nguyen A, Goujard C (2005). HIV controllers: a homogeneous group of HIV-1-infected patients with spontaneous control of viral replication.. Clin Infect Dis.

[14] Bristow J, Carey W, Egging D, Schalkwijk J (2005). Tenascin-X, collagen, elastin, and the Ehlers-Danlos syndrome.. Am J Med Genet C Semin Med Genet.

[15] Rupert KL, Moulds JM, Yang Y, Arnett FC, Warren RW (2002). The molecular basis of complete complement C4A and C4B deficiencies in a systemic lupus erythematosus patient with homozygous C4A and C4B mutant genes.. J Immunol.

[16] White RA, Pasztor LM, Richardson PM, Zon LI (2000). The gene encoding TBC1D1 with homology to the tre-2/USP6 oncogene, BUB2, and cdc16 maps to mouse chromosome 5 and human chromosome 4.. Cytogenet Cell Genet.

[17] Suske G, Bruford E, Philipsen S (2005). Mammalian SP/KLF transcription factors: bring in the family.. Genomics.

[18] Turner J, Crossley M (1999). Basic Kruppel-like factor functions within a network of interacting haematopoietic transcription factors.. Int J Biochem Cell Biol.

[19] Dominguez A, Ramos-Morales F, Romero F, Rios RM, Dreyfus F (1998). hpttg, a human homologue of rat pttg, is overexpressed in hematopoietic neoplasms. Evidence for a transcriptional activation function of hPTTG.. Oncogene.

[20] Prezant TR, Kadioglu P, Melmed S (1999). An intronless homolog of human proto-oncogene hPTTG is expressed in pituitary tumors: evidence for hPTTG family.. J Clin Endocrinol Metab.

[21] Chun TW, Justement JS, Lempicki RA, Yang J, Dennis G (2003). Gene expression and viral prodution in latently infected, resting CD4+ T cells in viremic versus aviremic HIV-infected individuals.. Proc Natl Acad Sci U S A.

[22] Xu J, Wu H, Zhang C, Cao Y, Wang L (2002). Identification of a novel human DDX40gene, a new member of the DEAH-box protein family.. J Hum Genet.

[23] Jeang KT, Yedavalli V (2006). Role of RNA helicases in HIV-1 replication.. Nucleic Acids Res.

[24] Hosono K, Sasaki T, Minoshima S, Shimizu N (2004). Identification and characterization of a novel gene family YPEL in a wide spectrum of eukaryotic species.. Gene.

[25] Bobardt MD, Salmon P, Wang L, Esko JD, Gabuzda D (2004). Contribution of proteoglycans to human immunodeficiency virus type 1 brain invasion.. J Virol.

[26] Bobardt MD, Saphire AC, Hung HC, Yu X, Van der SB (2003). Syndecan captures, protects, and transmits HIV to T lymphocytes.. Immunity.

[27] Ngo-Giang-Huong N, Deveau C, Da SI, Pellegrin I, Venet A (2001). Proviral HIV-1 DNA in subjects followed since primary HIV-1 infection who suppress plasma viral load after one year of highly active antiretroviral therapy.. AIDS.

[28] Hao K, Schadt EE, Storey JD (2008). Calibrating the performance of SNP arrays for whole-genome association studies.. PLoS Genet.

[29] de Bakker PI, McVean G, Sabeti PC, Miretti MM, Green T (2006). A high-resolution HLA and SNP haplotype map for disease association studies in the extended human MHC.. Nat Genet.

[30] Liu Y, Helms C, Liao W, Zaba LC, Duan S (2008). A genome-wide association study of psoriasis and psoriatic arthritis identifies new disease Loci.. PLoS Genet.

[31] Magierowska M, Theodorou I, Debre P, Sanson F, Autran B (1999). Combined genotypes of CCR5, CCR2, SDF1, and HLA genes can predict the long-term nonprogressor status in human immunodeficiency virus-1-infected individuals.. Blood.

[32] Gillespie GM, Kaul R, Dong T, Yang HB, Rostron T (2002). Cross-reactive cytotoxic T lymphocytes against a HIV-1 p24 epitope in slow progressors with B*57.. AIDS.

[33] Schneidewind A, Brockman MA, Sidney J, Wang YE, Chen H (2008). Structural and functional constraints limit options for cytotoxic T-lymphocyte escape in the immunodominant HLA-B27-restricted epitope in human immunodeficiency virus type 1 capsid.. J Virol.

[34] Fodil N, Laloux L, Wanner V, Pellet P, Hauptmann G (1996). Allelic repertoire of the human MHC class I MICA gene.. Immunogenetics.

[35] Pellet P, Renaud M, Fodil N, Laloux L, Inoko H (1997). Allelic repertoire of the human MICB gene.. Immunogenetics.

[36] Bauer S, Groh V, Wu J, Steinle A, Phillips JH (1999). Activation of NK cells and T cells by NKG2D, a receptor for stress-inducible MICA.. Science.

[37] Groh V, Rhinehart R, Randolph-Habecker J, Topp MS, Riddell SR (2001). Costimulation of CD8alphabeta T cells by NKG2D via engagement by MIC induced on virus-infected cells.. Nat Immunol.

[38] Mellors JW, Griffith BP, Ortiz MA, Landry ML, Ryan JL (1991). Tumor necrosis factor-alpha/cachectin enhances human immunodeficiency virus type 1 replication in primary macrophages.. J Infect Dis.

[39] Carrington M, Nelson GW, Martin MP, Kissner T, Vlahov D (1999). HLA and HIV-1: heterozygote advantage and B*35-Cw*04 disadvantage.. Science.

[40] Martin MP, Qi Y, Gao X, Yamada E, Martin JN (2007). Innate partnership of HLA-B and KIR3DL1 subtypes against HIV-1.. Nat Genet.

[41] Goujard C, Bomarek M, Meyer L, Bonnet F, Chaix ML (2006). CD4 cell count and HIV DNA level are independent predictors of disease progression after primary HIV type 1 infection in untreated patients.. Clin Infect Dis.

[42] Saez-Cirion A, Lacabaratz C, Lambotte O, Versmisse P, Urrutia A (2007). HIV controllers exhibit potent CD8 T cell capacity to suppress HIV infection ex vivo and peculiar cytotoxic T lymphocyte activation phenotype.. Proc Natl Acad Sci U S A.

[43] Kelley CF, Barbour JD, Hecht FM (2007). The relation between symptoms, viral load, and viral load set point in primary HIV infection.. J Acquir Immune Defic Syndr.

[44] Viard JP, Burgard M, Hubert JB, Aaron L, Rabian C (2004). Impact of 5 years of maximally successful highly active antiretroviral therapy on CD4 cell count and HIV-1 DNA level.. AIDS.

[45] Devlin B, Roeder K (1999). Genomic control for association studies.. Biometrics.

[46] Dalmasso C, Broet P, Moreau T (2005). A simple procedure for estimating the false discovery rate.. Bioinformatics.

[47] Sun L, Craiu RV, Paterson AD, Bull SB (2006). Stratified false discovery control for large-scale hypothesis testing with application to genome-wide association studies.. Genet Epidemiol.

[48] Guedj M, Wojcik J, Della-Chiesa E, Nuel G, Forner K (2006). A fast, unbiased and exact allelic test for case-control association studies.. Hum Hered.

[49] Clayton D, Leung HT (2007). An R package for analysis of whole-genome association studies.. Hum Hered.

[50] Boos DD (1992). On generalized score test.. Am Stat.

