# Supplementary figures and images for: Distinct Genetic Loci Control Plasma HIV-RNA and Cellular HIV-DNA Levels in HIV-1 Infection: The ANRS Genome Wide Association 01 Study
Source: PLoS One. 2008 Dec 24;3(12):e3907. doi: 10.1371/journal.pone.0003907 (PMC2603319; doi:10.1371/journal.pone.0003907)

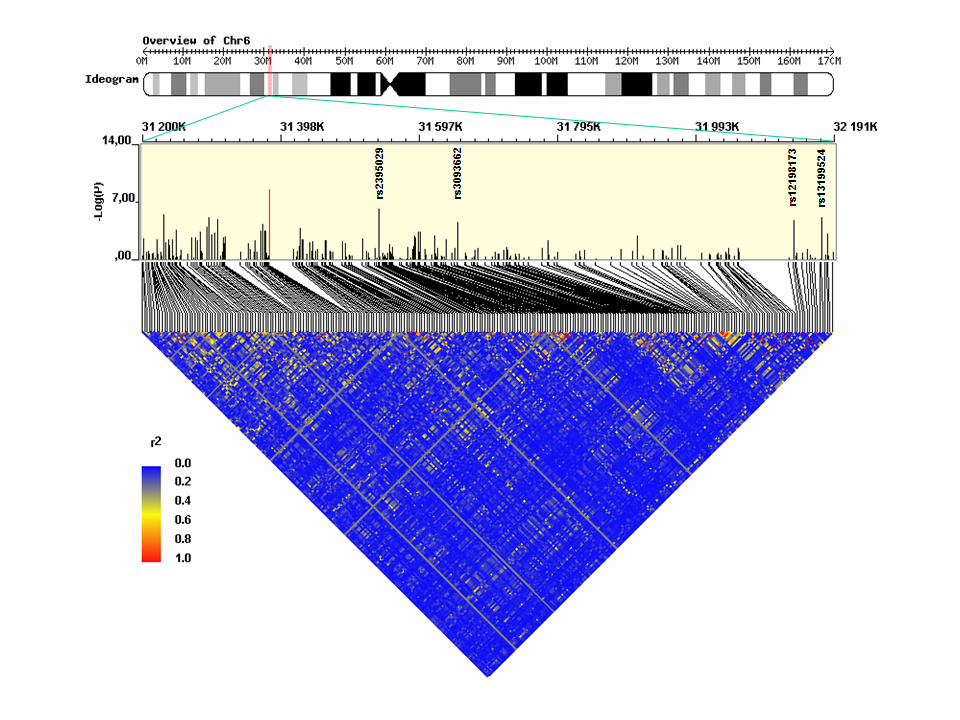

Supplement: Figure S1 — Upper box: An ideogram depicting chromosome 6 and the annotated region (transparent red rectangle). Middle box: show the SNPs with their associated -log10(P)-values, these lines are spaced according to their actual physical location. Positions of the four SNPs (rs2395029, rs13199524, rs12198173 and rs3093662) are indicated on the figure. Lower box: Linkage disequilibrium data matrix is based on HapMap CEU data. The figure has been constructed using WGAViewer software (http://people.genome.duke.edu/~dg48/WGAViewer/download.php, Ge D, Zhang K, Need AC, Martin O, Fellay J, Urban TJ, Telenti A, Goldstein DB. WGAViewer: software for genomic annotation of whole genome association studies.Genome Res. 2008, 18:640–643). (0.54 MB TIF) [file pone.0003907.s001.tif]

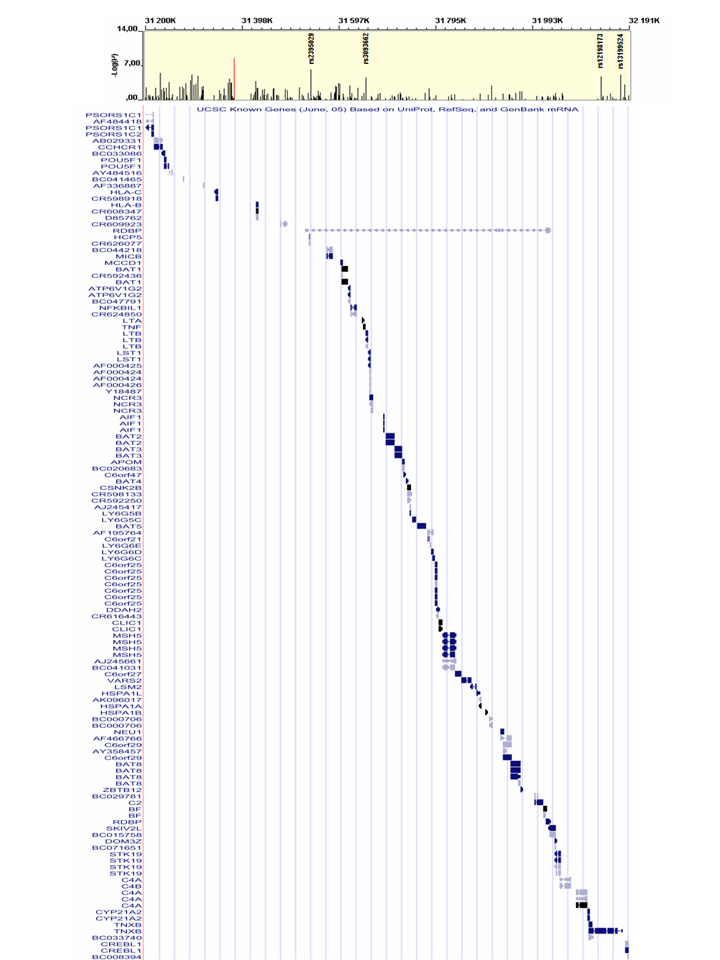

Supplement: Figure S2 — Upper box shows the SNPs with their associated -log10(P)-values, these lines are spaced according to their actual physical location. Positions of the four SNPs (rs2395029, rs13199524, rs12198173 and rs3093662) are indicated on the figure. Lower box shows the position of the UCSC known Genes (June, 05 and based on UniProt, RefSeq, and GenBank mRNA) located in the annotated region. The figure has been constructed using Genome Browser software, Kent WJ, Sugnet CW, Furey TS, Roskin KM, Pringle TH, Zahler AM, Haussler D. The human genome browser at UCSC. Genome Res. 2002,12: 996–1006). (0.42 MB TIF) [file pone.0003907.s002.tif]
